# PFAS Uptake by Plants in Soils Amended with Biosolids Derived from Wastewater with Industrial Input

**DOI:** 10.1021/acsenvironau.6c00004

**Published:** 2026-04-21

**Authors:** Aswin Kumar Ilango, Madhav Kharel, Weilan Zhang, Yanna Liang

**Affiliations:** Department of Environmental and Sustainable Engineering, 1084 University at Albany, State University of New York, Albany, New York 12222, United States

**Keywords:** biochar, biosolids, conservation reserve program, PFAS, phytoremediation

## Abstract

Per- and polyfluoroalkyl substances (PFAS) present in biosolids pose challenges for the management of biosolids land application. This study evaluated the phytoremediation potential of a grass-legume mixture derived from a Conservation Reserve Program (CRP) seed blend for removing PFAS from biosolids-amended soils treated with biochar. Thirteen representative PFAS were monitored in plant tissues over a 92 day greenhouse experiment. Results showed that PFAS uptake was significantly influenced by soil type, biochar type and dose, and biosolids characteristics. Notably, biosolids derived from a mixture of industrial and domestic wastewater, containing higher concentrations of PFAS precursors (1515.9 ± 1.89 μg/kg), resulted in reduced plant biomass and lower ΣPFAS removal of 0.3–0.7% compared to 1.0–2.5% of ΣPFAS removal with biosolids comprising PFAS precursors of 6.30 ± 1.89 μg/kg sourced solely from domestic wastewater. While low-dose biochar (0.05%) enhanced PFAS uptake under certain conditions, higher doses (5%) generally stabilized PFAS, reducing their bioavailability. Isotopically labeled PFHxS and PFNA confirmed these patterns, with chain length influencing uptake efficiency. Leaching tests using water revealed that biochar amendments reduced PFAS leachability in Scantic soil but increased it in Woodbridge soil, highlighting the need for site-specific remediation strategies. Overall, PFAS phytoextraction was less effective in biosolids derived from mixed industrial and domestic sources compared with those from domestic wastewater alone. This study highlights the importance of tailoring phytoremediation and biochar applications to biosolids composition and soil properties to ensure safe and effective PFAS management.

## Graphical abstract

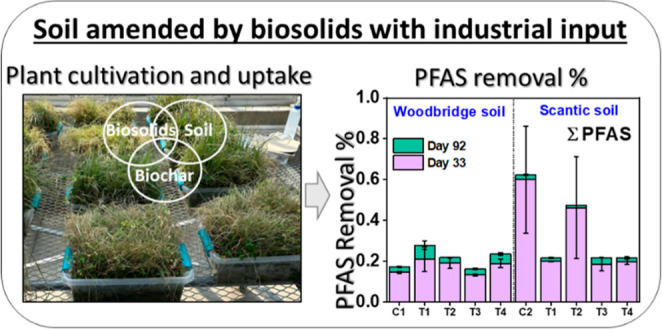

## 1 Introduction

Per- and polyfluoroalkyl substances (PFAS) comprise over 7 million organic compounds containing at least one saturated CF_2_ or CF_3_ moiety, according to the revised definition of the Organization for Economic Co-operation and Development (OECD).^1^ The strong carbon–fluorine bonds (115 kcal/mol), hydrophobic tail, and polar functional group in these molecules contribute to the unique properties of PFAS, including extreme chemical and thermal stability, hydrophobic and lipophobic behavior, and high surface activity.^2,3^ These properties have led to their widespread use in aqueous film-forming foams (AFFFs), packaging, textiles, cosmetics, coating materials, and food contact materials.^4–7^ However, PFAS, especially those with longer carbon chains, have been found to be potentially hazardous, carcinogenic, and prone to phytoaccumulation, posing health risks to humans through direct intake of PFAS-containing food and water.^8^

One of the primary pathways for PFAS entry into the food web is through the land application of biosolids produced at wastewater treatment plants (WWTPs).^9^ Due to the ineffectiveness of biological treatment processes commonly employed at WWTPs in degrading PFAS, biosolids, as a byproduct, have been recognized as a reservoir for legacy perfluoroalkyl acids (PFAAs), emerging alternatives, and PFAA precursors.^10–12^ The estimated annual load of ΣPFAS in U.S. biosolids reported in 2014 ranged from 2749 to 3450 kg, with 1375 to 2070 kg applied to agricultural land and 467 to 587 kg disposed of in landfills.^13^ Between 2007 and 2019, the Maine Department of Environmental Protection (DEP) collected over 30,000 records on the screening of 28 PFAS compounds at 245 sites across the state.^14^ The analysis revealed the presence of PFOA and PFOS in biosolids sampled from WWTPs at these sites, with average concentrations of 10.1 and 25.3 ng/g dry weight, respectively. After being applied to land, PFAS in the biosolids can be released to the soil, further migrating into nearby surface water or leaching into groundwater. From a remediation perspective, although soil washing^15^ and thermal treatment^16,17^ may work, they severely disrupt the soil structure, render the soil undesirable for growing crops, and are not suitable for agricultural soils. Therefore, less invasive and sustainable remediation strategies are urgently needed to mitigate PFAS’ long-term impact on the environment and human health while preserving the soil properties.

Recent research has reported the varying phytoaccumulation potential of PFAS across different plant species, including agricultural crops, wetland vegetation, and trees.^18–23^ Given these findings, phytoremediation presents a promising approach for mitigating PFAS contamination by stabilizing, accumulating, and facilitating their removal from contaminated water and soil.^24^ However, enhancing the efficiency of phytoremediation remains a key challenge, particularly in improving PFAS uptake by plants to maximize removal from the soil. One potential strategy is the use of biochar amendments. Our previous study demonstrated that biochar, when applied at an appropriate dose, can increase uptake of PFAS by timothy grass (TG).^25^

The findings with TG motivated further investigation into the interactions between biochar, plant species, and PFAS-contaminated biosolids, as illustrated in our recent publication reporting a grass-legume mixture.^26^ This grass-legume mixture is a Conservation Reserve Program (CRP) seed mix, a carefully selected blend of grasses and legumes designed for CRP-enrolled land based on local environmental conditions. The CRP, administered by the Farm Service Agency (FSA) under the U.S. Department of Agriculture (USDA), aims to restore valuable land cover, improve water quality, prevent soil erosion, and protect wildlife habitats.^27^ Our previous results showed that six representative PFAS compounds, including three perfluoroalkyl carboxylic acids (PFCAs) (C6, C7, and C8) and three perfluoroalkyl sulfonic acids (PFSAs) (C4, C6, and C8), were taken up and transferred to plant shoots with uptake patterns influenced by plant growth duration, biochar dose, and soil properties. Over time, upward translocation of short-chain PFAS became more dominant, with removal efficiencies ranging from 5% to 20%. A low biochar dose (0.05%) enhanced uptake, while a higher dose (1%) stabilized PFAS in soil, reducing plant uptake.^26^ These findings reinforce the feasibility of phytoremediation for PFAS removal. However, in this previous study,^26^ the biosolids were derived completely from municipal wastewater without any industrial inputs. The total concentration of PFAS precursors measured by a total oxidation precursor (TOP) assay was 6.30 ± 1.89 μg/kg. Thus, at this stage, it is uncertain whether a similar approach can be applied to biosolids originating from WWTP with industrial input. Biosolids receiving industrial inputs are known to contain unidentified PFAS precursors.^28,29^ For example, polyfluoroalkyl phosphate esters (PAPs), fluorooctane sulfonamides, sulfonamidoethanols, and sulfonamidoacetic acids (FOSA/E/AAs) have been reported by Moodie et al.^10^ These precursors are known to be transformable in soil with perfluoroalkyl acids (PFAAs) as the major terminal products.^30–32^ Given the possibility that different precursors may be transformed to similar PFAAs, we focused on the uptake and distribution of the end products of the PFAS precursors.

In this study, we further evaluated the phytoremediation potential of the same CRP plant mixture for removing PFAS in biosolids generated at a WWTP with industrial inputs. Similar to our previous study,^26^ this work also aimed to investigate the effect of different types of biochar, biochar dose, and different soil properties on the extent of PFAS removal. Additionally, comparison of results between the previous^26^ and this study was performed to gain critical insights into the practicality of using plant uptake for remediating soil contaminated by PFAS in biosolids. This direct comparison was made possible since these two studies were performed by the same researchers using the same types of soil, biochar, and CRP mix. The only difference between these two studies was the biosolids. Thus, the work we report here provides a unique assessment of the broader applicability of combining a biochar and CRP mix for treating agricultural soils contaminated by PFAS through biosolids application.

## 2 Materials and Methods

### 2.1 Soil Preparation

Table S1 lists all of the chemicals and reagents used in this study. Two soil types, Woodbridge and Scantic, representing soils in the state of Maine, USA, were collected from different locations in this state. Detailed characteristics of the two soils are given in Table S2. Biosolids used in this study were obtained from a WWTP in Maine as well. This WWTP receives an average of 30–40% of its annual wastewater flow from a paper products manufacturer. Background PFAS levels in the soils and biosolids were quantified using standard extraction and analysis methods detailed in EPA Method 1633.^33^ Two types of biochar were used: Black Owl Biochar Environmental Ultra (referred to as biochar #1 in the following, >85% organic carbon, ∼800 m^2^/g surface area) derived from forest wood waste and a custom-made biochar (referred to as biochar #2, 81% organic carbon, ∼63 m^2^/g surface area) produced from wood and grass.

The biosolids were spiked with 40 μg/kg (dry weight) of two isotopically labeled PFAAs (^13^C_3_-PFHxS and ^13^C_9_-PFNA), then mixed with biochar #1 or #2 at a low (0.05 wt %) or high (1 wt %) dose. The isotopically labeled PFAAs were used to track their distribution in the studied soil-biosolids-plant systems while minimizing interference from potential transformation of native PFAS precursors in the biosolids. The two doses of biochar were chosen considering the results we observed previously^26^ where biochar at 0.05% enhanced CRP’s uptake of PFAS, while 1% led to PFAS stabilization. Given the higher concentrations of PFAS precursors in the biosolids used in this study, a high biochar dose of 5% was selected.

The biochar-treated biosolids were then blended with sieved (2 mm) Woodbridge or Scantic soil. Each mixture containing 2.5 kg (dry weight) of soil and 0.25 kg (dry weight) of biochar amended biosolids was placed into 7-quart polypropylene containers (12 in. *L* × 6.5 in. *W* × 6″ H). This ratio of biosolids to soil aimed to achieve a biosolids application rate of 5 dry tons/acre, in accordance with EPA guidelines. Each soil type included five treatment groups: one control with biosolids but no biochar and four treatments in which biosolids were mixed with either biochar #1 or biochar #2 at the low or high dose.

### 2.2 Greenhouse Plant Cultivation

A CRP seed mixture containing four grass and five legume species at specific weight ratios (Table S3) was purchased from Millborn Seeds (Brookings, SD, U.S.). The CRP mixture consisted of four grass species (Kentucky Bluegrass, Timothy Grass, Orchard Grass, and Smooth Bromegrass) and five legume species (Alfalfa, Red Clover, White Clover, Alsike Clover, and Birdsfoot Trefoil). Prior to sowing, the CRP seed mixture was inoculated with *N*-Dure (Verdesian Life Sciences, Cary, NC, USA), a seed inoculant containing specialized *Rhizobia* bacteria to enhance nitrogen fixation and nodulation in leguminous species. The recommended amount of *N*-Dure was mixed with a small quantity of water to improve seed adhesion and applied according to the manufacturer’s guidelines at the time of planting. The treated seed mixture was then distributed at a rate of 10 g per container over the prepared soil. Following inoculation, each container received 800 mL of tap water and was placed in a greenhouse at the University at Albany. The use of tap water was to simulate realistic irrigation conditions, as agricultural and biosolids-amended systems are typically irrigated with municipal water rather than deionized (DI) water. The tap water was also analyzed for background PFAS, including the six representative compounds, and none of the target chemicals were detected above the method reporting limits, as shown in Table S4. Starting on Day 5, the germinated seedlings were watered daily to maintain soil moisture at 80% field capacity by keeping the container weight constant. On Day 33, the plant shoots from each container were cut, rinsed with deionized water, and processed for PFAS extraction. Plants were allowed to regrow under identical conditions in the greenhouse until Day 92, when shoots were harvested a second time for PFAS analysis. To compensate for seasonal reductions in natural light, artificial illumination (10 h per day) was started from Day 55 to ensure the plants had a light/dark cycle of 16/8. After the second harvest, the biosolids-amended soil in each container was air-dried in a fume hood for 2 weeks, ground into fine particles, and thoroughly mixed before leaching and extracting the remaining PFAS.

### 2.3 PFAS Extraction, Leaching, and Quantification

PFAS in biosolids and soil samples were extracted following EPA Method 1633 with minor modifications. In brief, 0.5 g of dried biosolids or dried soil was fortified with 10 ng of ^13^C_5_-PFHxA extracted internal standard (EIS) and placed in a 50 mL polypropylene tube containing 10 mL of 0.3% methanolic NaOH. After vortexing for 20 s and shaking at 150 rpm for 2 h, the mixture was centrifuged at 4500 rpm for 10 min, and the supernatant was transferred to a clean tube. The solid residue was extracted twice more with 15 and 5 mL of the same solvent. The three extracts were combined, passed through an ENVI-Carb cartridge, and evaporated under nitrogen at 55 °C until the volume was less than 1 mL. The extract was then diluted with 10 mL of reagent water and subjected to solid-phase extraction (SPE) using a HyperSep C18 cartridge. PFAS in the final eluate were quantified using an Agilent 6470 Triple Quadrupole liquid chromatography–mass spectrometry (LC–MS)/MS.

In this study, ^13^C_5_-PFHxA was selected as a representative midchain PFAS to monitor extraction efficiency, consistent with the approach applied in our previous batch analysis of biosolids^20^ in order to maintain methodological consistency. The recoveries obtained using this surrogate were compared with those from the isotopically labeled EIS mixture recommended in EPA Method 1633.^33^ As shown in Table S5, the recoveries of both ^13^C_5_-PFHxA and the Method 1633 EIS compounds for our target PFAS were close to 100% in quality control (QC) samples across different spike masses (ng). These comparable recoveries indicate that the use of ^13^C_5_-PFHxA provided a reliable estimation of PFAS recoveries in the tested biosolids in this work.

The plant shoots were immediately freeze-dried after harvest and processed with an MTBE-NaOH extraction method.^34^ In short, each dry tissue sample was spiked with 10 ng of ^13^C_5_-PFHxA as the EIS, treated overnight with 0.4 M NaOH to lyse the sample, and mixed with 0.5 M tetrabutylammonium hydrogensulfate (TBAHS) and 0.25 M Na_2_CO_3_ buffer. The mixture was then extracted three times with 5 mL of methyl *tert*-butyl ether (MTBE). The combined 15 mL of MTBE fraction was evaporated, reconstituted in 1 mL of methanol plus 9 mL of water, and subjected to SPE. The eluate was split in half: one portion was analyzed directly by LC-MS/MS for PFAS quantification, while the other underwent a TOP assay.

For the TOP assay, following the procedure reported,^35^ the solvent was first evaporated. The remaining residue was then dissolved in 6 mL of a solution containing 60 mM persulfate and 150 mM NaOH, and the mixture was heated at 85 °C for 6 h to oxidize PFAS precursors. After cooling, the solution was neutralized using concentrated HCl, subjected to SPE, and reanalyzed to quantify the increase in PFCAs, which was attributed to the transformation of precursor compounds and used to estimate the concentration of total PFAS precursors.

To evaluate the leachability of PFAS in biosolids-amended soil, we performed a water-leaching test of soil after the second CRP harvest, adopting the Australian Standard Leaching Procedure (ASLP) with slight modifications. Briefly, 1 g of the biosolids–amended soil was spiked with 10 ng of ^13^C_5_-PFHxA EIS and mixed with 10 mL of deionized water, vortexed for 20 s, and agitated at 150 rpm for 2 h. The mixture was centrifuged at 4500 rpm for 10 min, and the supernatant was collected as leachate, which was then subjected to SPE for subsequent LC-MS/MS analysis.

Prior to LC-MS/MS analysis, all samples were spiked with nonextracted internal standards (^13^C_4_-PFOA and ^13^C_4_-PFOS). Detailed information on instrument settings, calibration procedures, and quality assurance measures can be found in Text S1 and Table S6 and referenced studies. The physicochemical properties of PFAS quantified in this study are detailed in Table S7.

## 3 Results and Discussion

### 3.1 Complex Mixtures of PFAS in the Biosolids

The biosolids, sourced from a municipal WWTP that receives a substantial portion of its influent from the paper manufacturing industry, contained a mixture of PFAS compounds of 115 μg/kg (Σ_13_PFAS). Among the PFSAs, PFOS was the most abundant, with a concentration of approximately 50.52 μg/kg. For PFCAs, PFOA, PFNA, and PFDA were detected at concentrations ranging from 2.5 to 8.0 μg/kg. In addition to PFAAs, 6:2 FTS and *N*-EtFOSAA were measured at 3.78 ± 1.82 and 41.88 ± 7.58 μg/kg, respectively (Figure 1).

**Figure 1:**
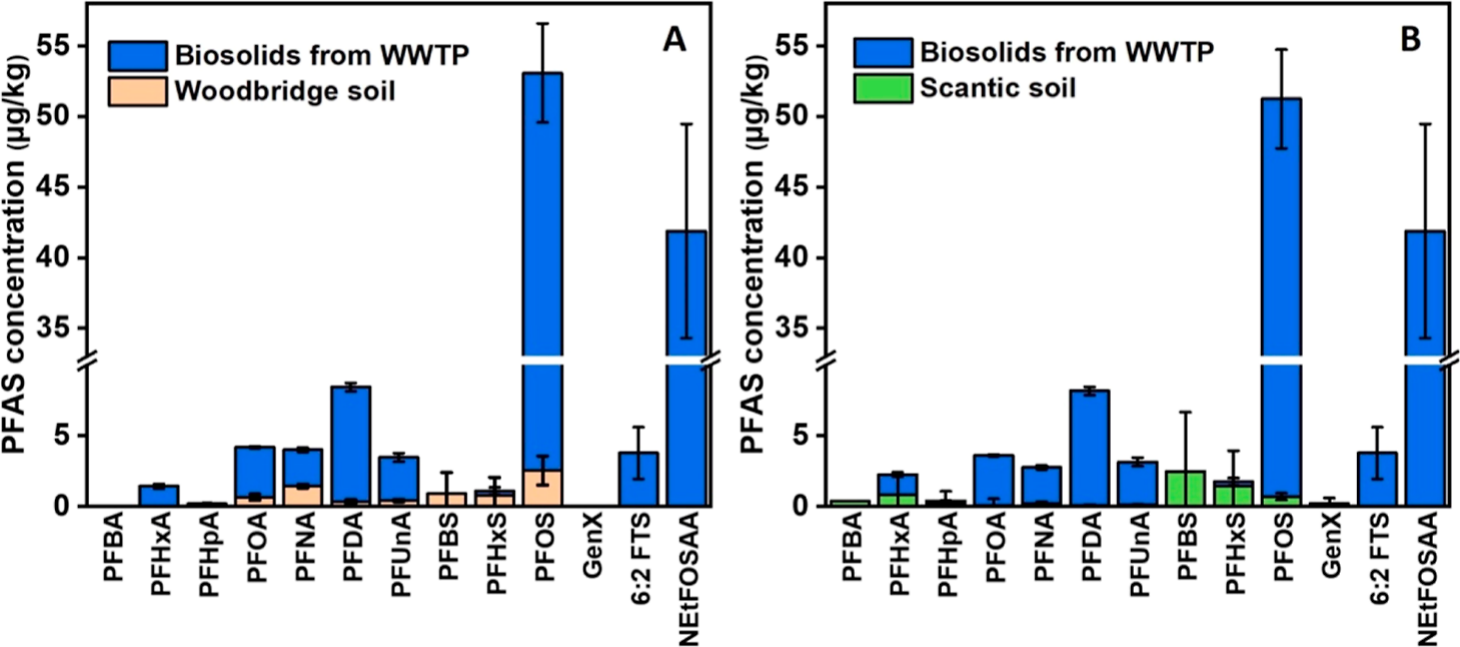
PFAS concentration (μg/kg) in the biosolids-amended Woodbridge or Scantic soil. Error bars represent the standard deviations of triplicate measurements. Each biosolids amended soil (BAS) mixture contained 2.5 kg (dry weight) of soil and 0.25 kg (dry weight) of biosolids, corresponding to a biosolids application rate of 5 dry tons/acre, in accordance with US EPA guidelines.

The TOP analysis revealed a total PFAS precursor concentration of 1515.9 ± 1.89 μg/kg. This method relies on heat-activated persulfate under alkaline pH of >12 to generate hydroxyl radicals, which oxidize precursor C–H bonds into terminal PFCAs.^36^ However, given the drawbacks of this assay, such as precursors lacking C–H bonds that may not be effectively converted, the transformation products that are not necessarily PFCAs, and the concentration of persulfate or the base that may not be optimal for complete conversion of all precursors, this analytic procedure potentially leads to an underestimation of total precursor burden in the biosolids tested.^36^ Although the Σ_13_PFAS concentration of 115 μg/kg biosolids used in this study was comparable to that of typical biosolids derived from domestic wastewater, as reported in our previous work (105 μg/kg),^26^ the total PFAS precursor concentration was >250-fold higher than what we observed for typical biosolids in our previous study. The significantly higher precursor concentrations were likely due to the contribution of industrial wastewater. PFAS and their precursors are commonly found in wastewater from the paper manufacturing industry due to their use as surface treatment agents that provide water, oil, and grease resistance to paper products, including food packaging and industrial materials.^37–39^ Thus, to ensure low concentrations of PFAS in WWTP’s effluent and biosolids, wastewater released from paper manufacturing facilities should be pretreated first to remove or at least decrease the PFAS load before being discharged to municipal WWTP.

### 3.2 Growth of the CRP Plant Mixture and Plant Uptake of PFAS over 92 days

#### 3.2.1 Dry Biomass of Plants

Figures S1 and S2 present the growth trajectory of the CRP plant mixture cultivated in biosolids-amended soils over a 92 day period. Germination began 1 week after sowing the seeds. Without biosolids, the two soils yielded a similar biomass weight of 6–7 g per container after 92 days (Figure S2A,B). This is similar to what we observed in our previous experiment using the same CRP mix,^26^ demonstrating that our experimental procedures and sample analysis are repeatable. For the Woodbridge soil, the biomass weight was not significantly enhanced by the target biosolids and biochar #2 at 0.05% (6.44 g). The other three treatments: biochar #1 at both 0.05% and 5% and biochar #2 at 5% did result in a much higher biomass yield in the range of 8.52–10.06 g. In terms of the Scantic soil, the amendment of biosolids alone led to a significant increase in biomass (12.68 g). Further addition of biochar, however, decreased the benefit of the biosolids by leading to lower biomass weight, i.e., 7.09–10.75 g. The reduced biomass in the biochar-biosolids treatment may result from nitrogen immobilization and nutrient sorption by biochar, which decreased plant-available nutrients and attenuated the fertilization effect of biosolids.^40,41^

It should be noted that biomass measurements were obtained from single containers per treatment. This considered the large surface area needed to grow the CRP and the complexity of setting up and maintaining biosolids-amended systems. As discussed above, given the repeatable results of biomass dry weight from controls without biosolids amendment between our previous^26^ and this current study, the biomass data, even from one single container, were reliable.

The biomass weights associated with biosolids amendment in Figure S2A are at least 2.7-fold lower than what we reported previously (Figure S2B) regarding the amendment of biosolids derived from only domestic wastewater, which is referred to as biosolids #1 for ease of discussion. The biosolids used in this study are referred to as biosolids #2 hereafter. With biosolids #1, the biomass yield after 92 days was 20–33 g per container. Thus, the beneficial effect of biosolids in enhancing plant growth is dependent on the characteristics of the biosolids. Our results indicate that biosolids with industrial relevance have a less beneficial effect than those without. This could be due to the presence of high concentrations of PFAS precursors or other unknown components tied to industrial input.

#### 3.2.2 Uptake of Dominant PFAS

Figure 2 shows the percent removal of dominant and EPA-regulated PFAS detected in the prepared biosolids-amended soils (i.e., PFOA, PFNA, and PFOS) by plants. Although *N*-EtFOSAA was detected at 42 μg/kg in biosolids #2, it was absent in the collected biomass, possibly due to its relatively high hydrophobicity, which led to undetectable upward translocation to plant shoots. Moreover, *N*-EtFOSAA may undergo rapid transformation into metabolites such as *N*-EtFOSA, FOSA, FOSAA, and the stable end product PFOS, further reducing its translocation.^42^ Wen et al.^43^ also reported high concentrations of *N*-EtFOSAA (150.93 ng/g) in biosolids-amended soils but negligible accumulation in plants, including alfalfa, lettuce, maize, mung bean, radish, ryegrass, and soybean. This limited translocation was attributed to both the rapid transformation of *N*-EtFOSAA within just 10 days of plant growth and its hydrophobicity, which favors retention in root tissues over transfer to aboveground biomass.

**Figure 2:**
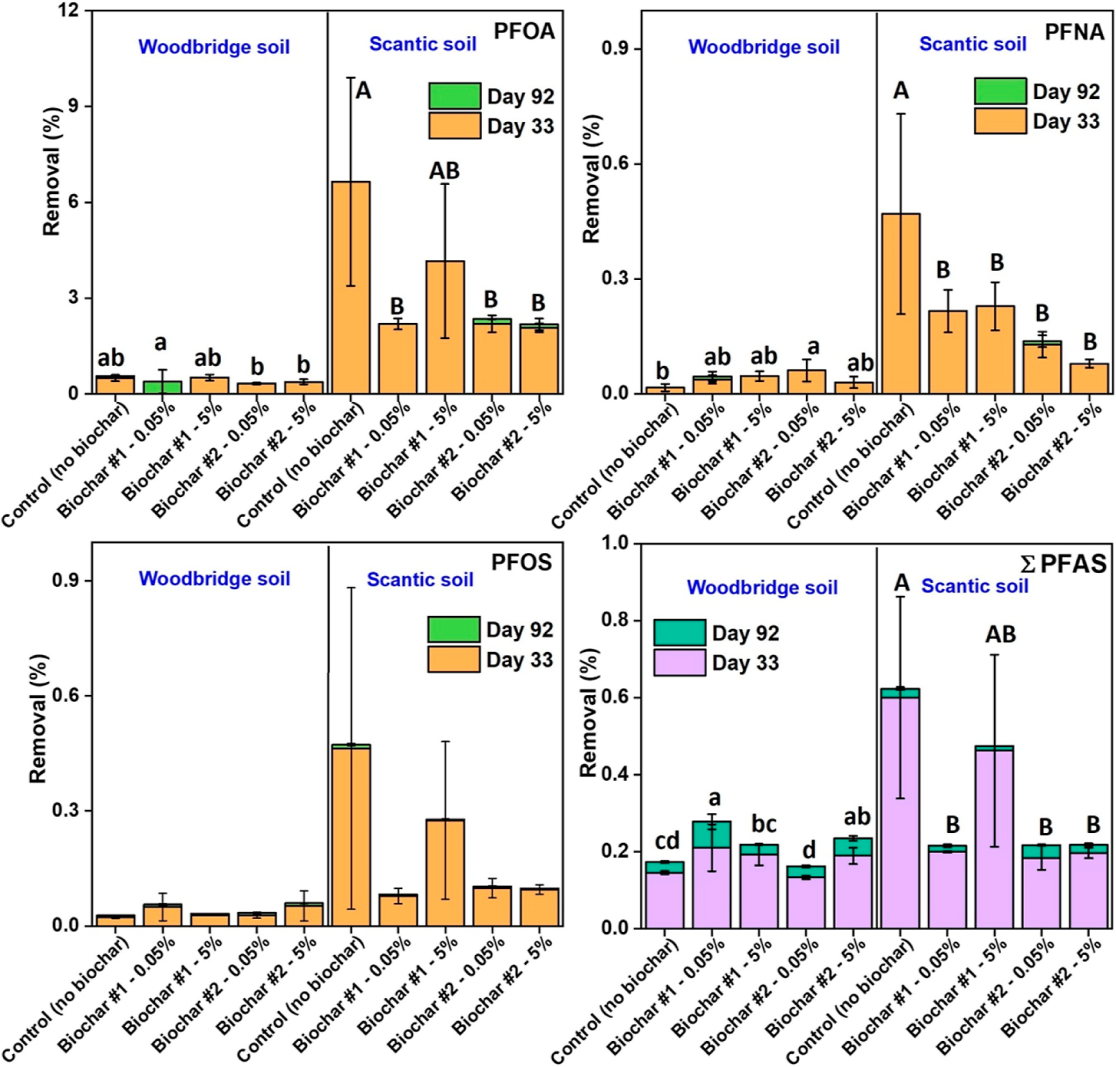
Removal efficiency (%) of representative and ΣPFAS compounds by CRP grass mixture harvested on Day-33 and Day-92 and combined removal over 92 days (*n* = 3). Different letters in lower case and upper case represent significant differences among the treatments with Woodbridge soil and Scantic soil, respectively (*p* < 0.05). Removal efficiency (%) was calculated as the ratio of the total mass of PFAS accumulated in plant tissues (ng) to the total mass of PFAS present in the BAS mixture (ng), multiplied by 100.

Notably, the removal of PFOA, PFNA, and PFOS between Day 33 and Day 92 was significantly lower than that during the first 33 days, suggesting that plant uptake of these long-chain compounds was substantially reduced following the first harvest. This trend closely aligns with our previous study using biosolids #1, where PFOA and PFOS were initially detected in CRP shoots at the first harvest (Day 33) but decreased 2–10-fold as the growth period extended to Day 93.^26^ Even during the first 33 days, the exact time effect on plant uptake of these PFAS is unclear. For example, given the lack of time series data, it is unknown whether PFAS’ upward movement was fast at the beginning and then slowed down, or they had a constant rate during the time before the first harvest. Getting the time series data will not be simple and straightforward as real-time and nondestructive measurements of PFAS in plant tissues are not available. Having these data, however, would be invaluable, and further research is warranted, as it would allow us to develop strategies to maximize PFAS uptake by plants.

One-way and two-way ANOVA analyses (Table S8) revealed that both the biochar type and dose significantly affected the removal of these representative PFAS compounds in the two soil types. Specifically, in Woodbridge soil, biochar #2 at a 0.05% dose significantly enhanced PFNA removal. In Scantic soil, both biochar #1 and biochar #2, at both low and high doses, significantly reduced PFNA uptake by plants. Similarly, biochar #1 at 0.05% and biochar #2 at both doses significantly decreased plant uptake of PFOA. Neither biochar had a statistically significant effect on the removal of PFOS. Overall, both biochars #1 and #2 demonstrated limited potential to substantially enhance plant-assisted removal of the three dominant PFAS in either soil. Instead, the treatments appeared to stabilize PFOA and PFNA in the biosolids-amended soils, reducing their phytoavailability. The industrially influenced biosolids likely contain higher levels of PFAS precursors that could continuously transform in soil into terminal PFAAs, reducing apparent removal efficiency and plant biomass. However, the complexity of biosolids, unknown PFAS precursors, and the lack of analytical standards prevent identification of specific precursor-to-PFAA transformation pathways.

The percent removal of Σ_13_PFAS compounds quantified in this study, including PFBA, PFHxA, PFHpA, PFOA, PFNA, PFDA, PFUnA, PFBS, PFHxS, PFOS, GenX, 6:2 FTS, and *N*-EtFOSAA, is shown in Figure 2. Two-way ANOVA indicated that both biochar types and their interaction with biochar dose significantly affected Σ_13_PFAS removal in Woodbridge soil, whereas in Scantic soil, only the biochar dose showed a significant effect (Table S7). Specifically, one-way ANOVA revealed that biochar #1 at 0.05% and biochar #2 at 5% yielded the greatest enhancement in Σ_13_PFAS removal in Woodbridge soil. In contrast, no improvement was observed in Scantic soil; rather, biochar #1 at 0.05% and biochar #2 at both doses significantly reduced Σ_13_PFAS removal. In summary, only biochar #1 at 0.05% and biochar #2 at 5% showed potential for enhancing Σ_13_PFAS removal through plant uptake in Woodbridge soil.

The impact of biochar on contaminant remediation has been reported to be dose-dependent. At low doses (0.2–2%), biochar has been shown to increase PFAS uptake in timothy grass and CRP plants.^25,26^ However, in most cases, biochar application stabilizes PFAS in soils, thereby restricting their transfer to plants and aquatic systems.^44,45^ This outcome is consistent with the strong sorption capacity of biochar, which can sequester long-chain PFAS such as PFOA, PFOS, and PFNA through hydrophobic interactions, reducing their phytoavailability. Supporting this, Holly et al.^46^ demonstrated that biochar-biosolids coapplication reduced PFAS migration in leaching-prone soils, lowering concentrations of Σ_28_PFAS concentration, including C7–C10 PFCAs and C4–C8 PFSAs, by 40–64%. In contrast, short-chain PFAS, due to their higher solubility and mobility, were less strongly bound to biochar in complex soil systems.

#### 3.2.3 Uptake of Isotopically Labeled PFAS

To assess the distribution of PFAS in the biosolids-soil-plant system while eliminating potential interference from precursor transformation, ^13^C isotopically labeled PFHxS and PFNA were spiked into the biosolids at 40 μg/kg and subsequently tracked throughout the system. As shown in Figure 3, both isotopically labeled compounds were detected in plant tissues and exhibited a clear chain-length effect, with ^13^C_3_-PFHxS accumulating at significantly higher concentrations and higher uptake percentages in the CRP plants than ^13^C_9_-PFNA. Two-way ANOVA revealed that in Woodbridge soil, biochar type, dose, and their interaction significantly influenced the uptake of both isotopically labeled PFHxS and PFNA (Table S7). In Scantic soil, all three factors significantly affected the removal of isotopically labeled PFHxS, while only biochar dose had a significant impact on isotopically labeled PFNA. One-way ANOVA further showed that in Woodbridge soil, biochar #1 at 0.05% was the most effective in enhancing the removal of both isotopically labeled compounds. Both biochars #1 and #2, at both doses, also significantly improved the removal of labeled PFNA. In Scantic soil, biochar #2 at 0.05% showed the greatest enhancement in the removal of isotopically labeled PFHxS. Both biochars #1 and #2, at both doses, significantly reduced the removal of isotopically labeled PFNA, consistent with observations for native PFNA in the biosolids-amended Scantic soil. In summary, the biochar treatment showed potential for enhancing PFAS removal in Woodbridge soil, while in Scantic soil it more notably contributed to PFAS stabilization by reducing their phytoavailability.

**Figure 3:**
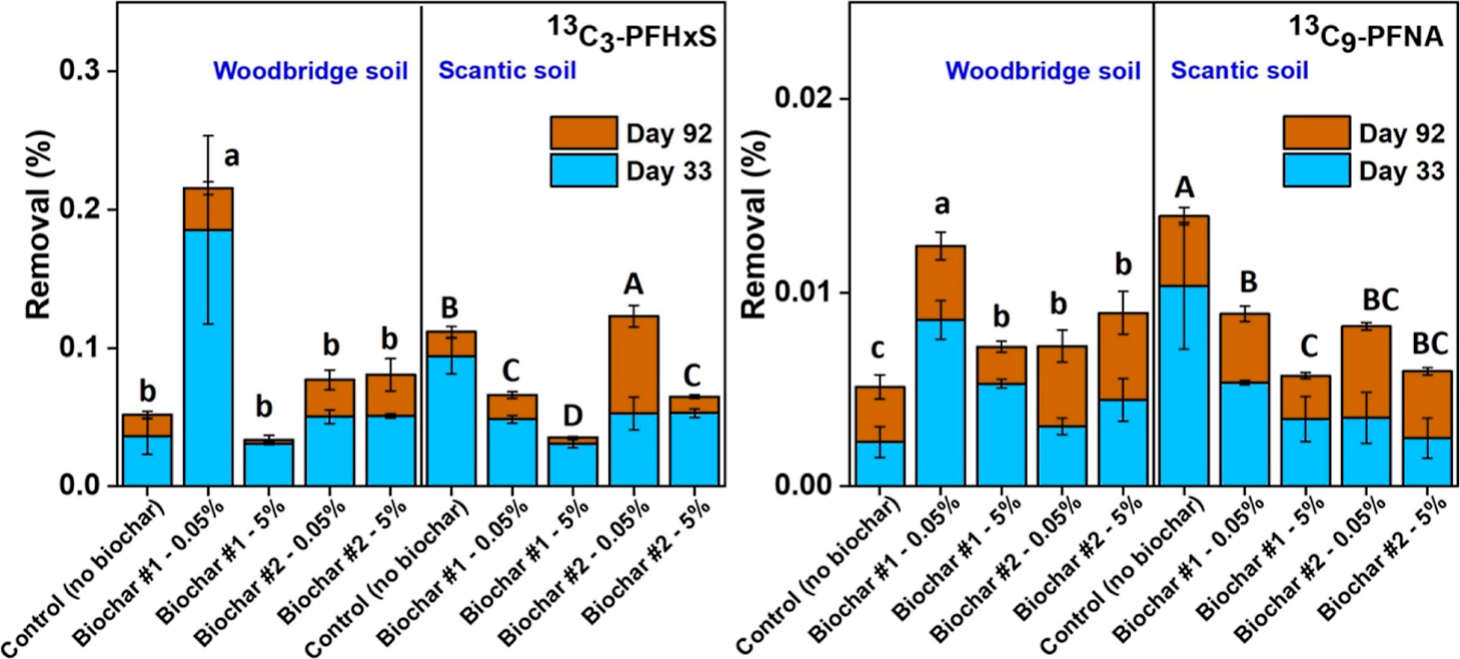
Removal efficiency (%) of isotopically mass-labeled PFHxS and PFNA by the CRP mixture harvested on Day-33 and Day-92 and combined removal over 92 days (*n* = 3). Different letters in lower case and upper case represent significant differences among the treatments with Woodbridge soil and Scantic soil, respectively (*p* < 0.05). Removal efficiency (%) was calculated as the ratio of the total mass of ^13^C-PFAS accumulated in plant tissues (ng) to the total mass of ^13^C-PFAS present in the BAS mixture (ng), multiplied by 100.

The rationale for spiking these two ^13^C-PFAS to soil was to enable an accurate mass balance of these two PFAAs without interference from the potential degradation of precursors in biosolids #2. While it is recognized that C_3_, C_4_, and crassulacean acid metabolism (CAM) photosynthetic pathways discriminate against ^13^CO_2_ during photosynthesis,^47^ the differential uptake of native vs ^13^C-PFAS by the CRP seed mix was unknown. In our previous study involving biosolids #1,^26^ the difference between the uptake percentage of PFHxS and ^13^C-PFHxS was not too significant. In this study, however, the difference for PFNA between the native and ^13^C labeled was drastically different (Figures 2 and 4). The significantly higher uptake of native PFNA could be explained by the following: (1) the CRP mix, mainly timothy grass, a C3 plant at the later growth phase, preferred translocation of native PFNA, and (2) the uptake of native PFNA was overestimated due to potential formation of this compound from PFAS precursors. At this point, due to the inability to track the extent of precursor transformation in the tested system, the exact explanation remains unknown. However, clearly, a better way to measure plant uptake of PFAS, including unknown precursors, is urgently needed.

**Figure 4:**
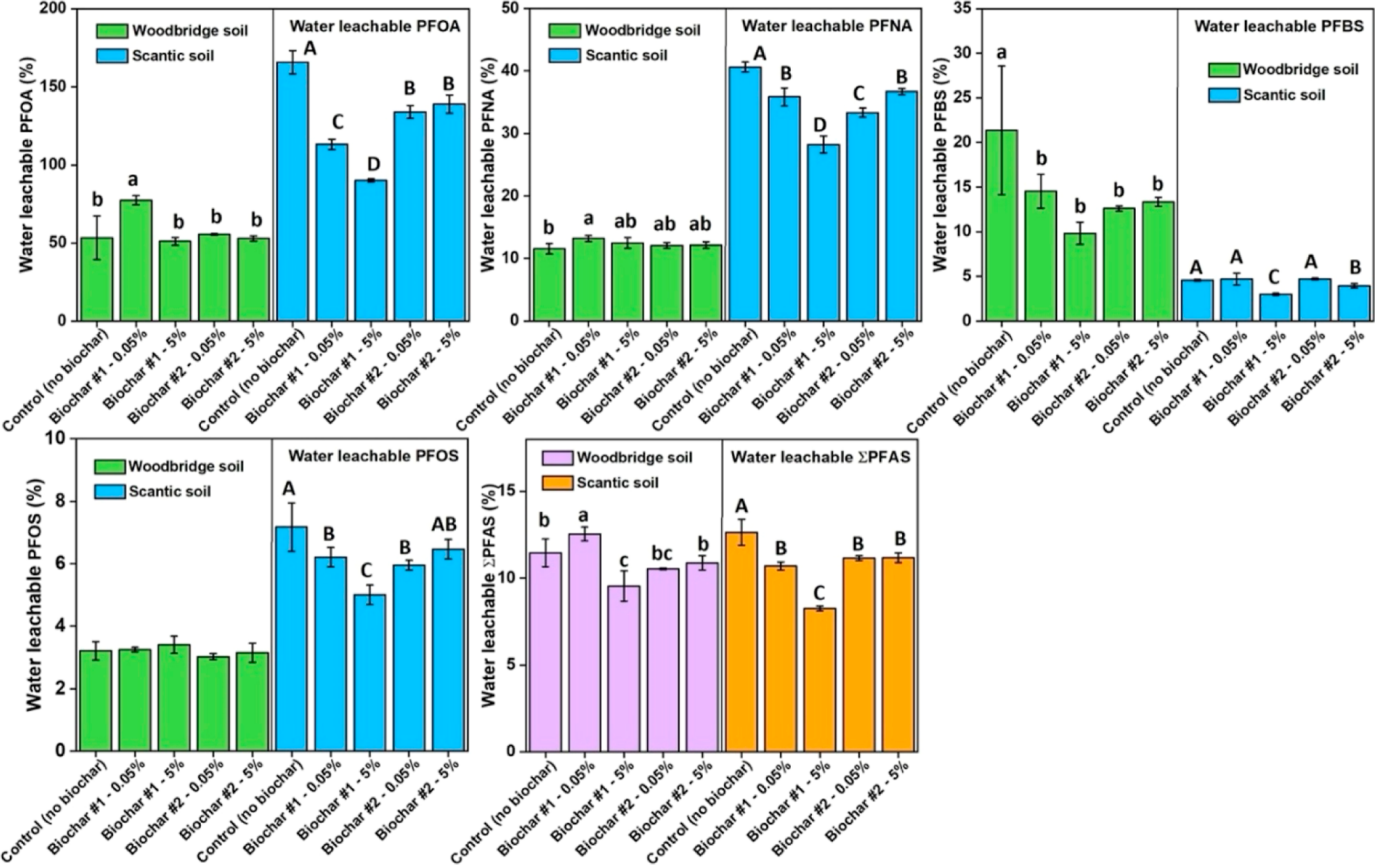
Water-leachable percentages of 4 representative PFAS, and ΣPFAS in soil on Day-92 (*n* = 3). Different letters in lower case and upper case represent significant differences among the treatments with Woodbridge soil and Scantic soil, respectively (*p* < 0.05). The water-leachable percentage (%) was calculated as the mass of PFAS leached (ng) divided by the total mass of PFAS present in the BAS mixture (ng) minus the mass accumulated in plant tissues (ng), then multiplied by 100.

### 3.3 Comparison of Σ_13_PFAS Removal by the CRP Mixture in Soil Amended by Two Types of Biosolids

Compared to our previous study using biosolids #1 from a WWTP receiving primarily domestic wastewater, the overall mass transfer of PFAS from soil to plant biomass was significantly lower in soils amended with biosolids #2 used in this study. At the time of the first harvest, the PFAS mass (ng) taken up by the CRP plant mixture exposed to biosolids #2 was approximately 50% of that measured in our previous work (Figure S3). During the second growth period from day 33 to day 92, the CRP plant mixture in this study exhibited substantially lower PFAS uptake (0.92–1.68 ng) compared to that in the first 33 days (5.02–12.73 ng). This was not consistent with our previous observations that mowing and regrowth promote further uptake. Biosolids #2 has been shown to contain a much larger fraction of PFAS in the form of precursors, substantially higher than the levels quantified in our older studies. Industrially sourced biosolids also typically contain complex mixtures of cocontaminants, including heavy metals, organic pollutants, and other PFAS variants. The high loading of these contaminants could interfere with plant physiology; for example, such toxicants may impair root growth^48,49^ or alter membrane transporter expression,^50–52^ both of which could reduce PFAS uptake. Moreover, PFAS uptake was positively correlated with dry biomass yield. By day 92, biomass production was reduced to at least half of that observed during the first 33 days (Figure S2A) and was 2.7-fold lower than in our previous study with biosolids #1 (Figure S2B). This sharp decline was likely caused by a visible insect infestation that appeared after day 50 in all biosolids #2-amended containers but not in the control, where biomass yields remained stable. The infestation may have been triggered by the complex composition of the biosolids with an industrial input. Consequently, differences in contaminant profiles and biosolids quality could plausibly explain the divergence in results between the present and previous studies.

Since the same types of soil, biochar, and CRP plant mixture were used in both studies over the same time frame, the observed differences in PFAS uptake, in terms of both percent removal and total PFAS mass, can be attributed to a single variable: the biosolids used. While the two biosolids types had comparable concentrations of PFAAs, they differed markedly in their concentrations of PFAA precursors. In our previous study, the total precursor concentration in biosolids #1 was 6.3 μg/kg, whereas the biosolids #2 used in the current study contained a much higher total precursor concentration of 1515.9 μg/kg. Research on the phytotoxicity of PFAS precursors to plants is still limited, but emerging evidence suggests that these compounds can cause physiological and biochemical stress, especially when plant antioxidant defense systems are overwhelmed.^53–55^ The transformation of PFAS precursors into more stable PFAAs in the biosolids-amended soils may further contribute to plant stress and reduced uptake. Additionally, PFAS precursors may indirectly affect plant health by disrupting soil microbial communities and rhizosphere processes, potentially reducing nutrient availability and altering PFAS mobility.^56,57^ Moreover, pollutants commonly found in paper manufacturing wastewater and the resulting biosolids can be toxic to plants and cause various forms of plant stress, both directly and indirectly.^58–60^ These stress responses and microbial disruptions could suppress root growth and metabolic activity, thereby influencing PFAS uptake dynamics and potentially explaining the lower uptake observed when the CRP plant mixture was exposed to higher concentrations of PFAS precursors.

Long-chain PFAS precursors and their derivatives may strongly bind to root tissues such as xylem due to their relatively high hydrophobicity, thereby limiting their upward translocation.^61^ Thus, to accurately characterize PFAS distribution, extraction from root tissues would have been essential; however, this was not performed in this work because the CRP grass–legume roots were extremely fine and difficult to separate from the complex soil matrix. Future investigations should therefore focus on mass balance analyses of PFAS within the entire soil-plant system to better understand the fate and distribution of PFAS precursors and their transformation products.

Combining the results of biomass dry weights and PFAS removal by the CRP plant mixture, it can be concluded that plant uptake, at least by the CRP seed mix, may not be a promising strategy for removing PFAS associated with biosolids #2 used in this study. These findings suggest that PFAS phytoextraction is not universally applicable across all sludge types. For biosolids with low ΣPFAS concentrations, including PFAS precursors, phytoremediation over time could be a viable approach. For those with a high concentration of PFAS and their precursors, however, plant uptake may be less ideal. Given that PFAS levels in most agricultural soils are typically in the tens and hundreds of μg/kg, phytoremediation remains a promising solution, though the exact rate and efficiency of plant uptake warrant further investigations.

### 3.4 Water-Leachable PFAS in the Biosolids-Amended Soil Systems

To assess the impact of biochar amendments on PFAS leachability in biosolids-soil-plant systems, a series of water leaching tests were conducted following the ASLP method after the second harvest of the CRP plants. Figure 4 presents the water-leachable percentages of the four EPA-regulated PFAS (i.e., PFOA, PFNA, PFBS, and PFOS) and ΣPFAS that were detectable in the biosolids-amended soils. It needs to be noted that while PFBS’s concentration in biosolids #2 was less than the limit of detection (LOD) of 0.005 μg/kg, it was quantified at 0.90 and 2.48 μg/kg in Woodbridge and Scantic soil, respectively. Thus, it was not surprising to see its appearance in the water leachate. On the contrary, while PFHxS was measured at 0.30 μg/kg in biosolids #2, 0.79 μg/kg in Woodbridge soil and 1.45 μg/kg in Scantic soil, its concentration was less than LOD of 0.02 μg/kg in the water leachate. The presence and absence of these two PFSAs in the water leachate could be due to their high and low water solubility, respectively. Additionally, a possible precursor transformation to PFBS cannot be excluded.

One-way ANOVA revealed that both types of biochar significantly reduced the water-leachable concentrations of PFOA, PFNA, PFBS, and PFOS in Scantic soil, with biochar #1 at 5% demonstrating the most effective stabilization performance. In contrast, in Woodbridge soil, only the PFBS leachability was reduced by biochar treatment. Notably, biochar #1 at 0.05% increased the leachability of PFOA and PFNA. It is worth mentioning that the leachable percentage of PFOA in Scantic soil exceeded 100%, which may be attributed to the possible transformation of PFAS precursors present in the system. Figure 5 also illustrates the leachable fractions of isotopically labeled PFHxS and PFNA. In Scantic soil, both types of biochar effectively reduced the water leachability of these mass-labeled compounds, with biochar #1 at 5% again showing the best performance. In contrast, no significant stabilization effect was observed in the Woodbridge soil. In fact, biochar #1 at 0.05% increased the leachability of both labeled PFHxS and PFNA. The total water-leachable Σ_13_PFAS from each treatment shown in Figure 5 also confirmed the patterns observed for both native and labeled compounds. In Scantic soil, both types of biochar significantly reduced total PFAS leachability, with biochar #1 at 5% achieving the greatest reduction. However, in the Woodbridge soil, biochar amendments showed no significant stabilization effect.

**Figure 5:**
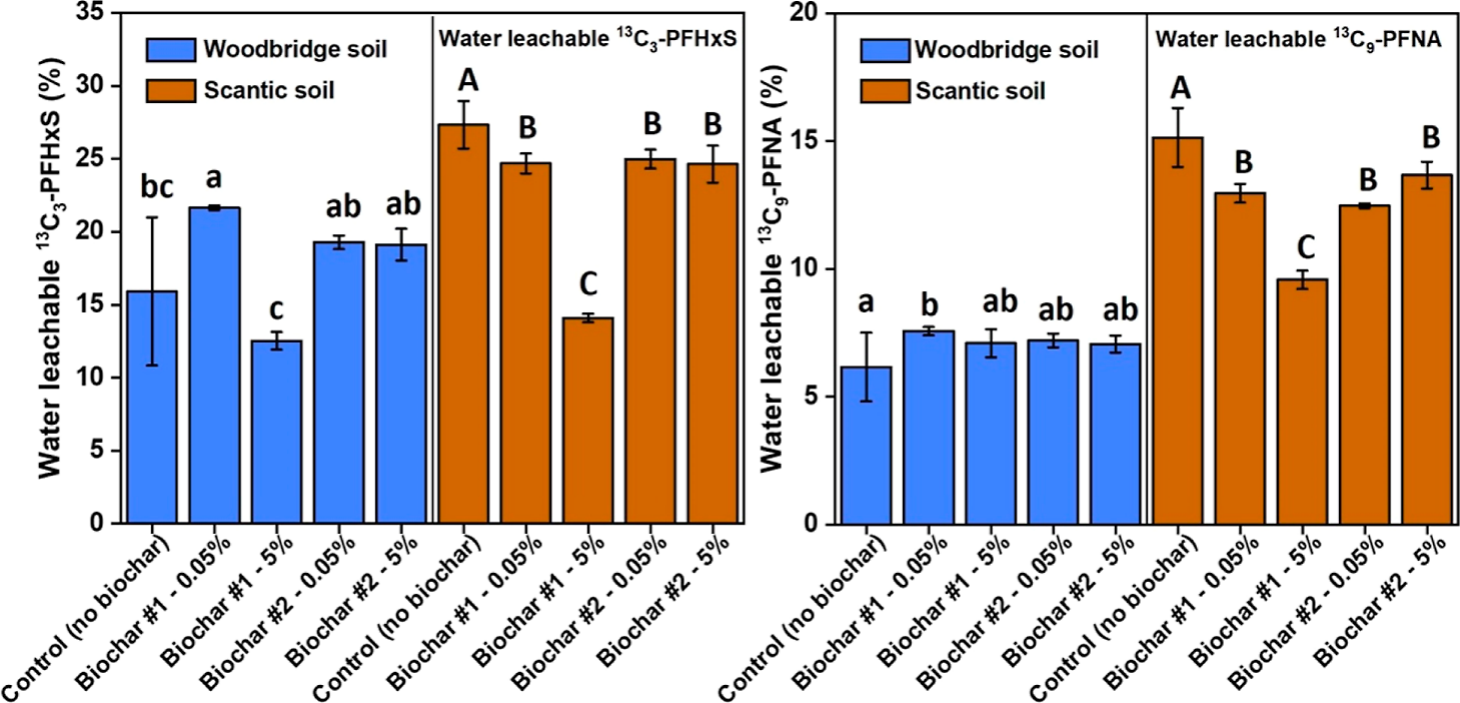
Water-leachable percentages of isotopically mass-labeled PFAS in soil on Day-92 (*n* = 3). Different letters in lower case and upper case represent significant differences among the treatments with Woodbridge soil and Scantic soil, respectively (*p* < 0.05). The water-leachable percentage (%) was calculated as the mass of ^13^C-PFAS leached (ng) divided by the total mass of ^13^C-PFAS present in the BAS mixture (ng) minus the mass accumulated in plant tissues (ng) and then multiplied by 100.

Although soil type may influence variations in PFAS binding and leaching, amendments such as biochar appear to play an important role in stabilizing a complex PFAS mixture. Similarly, Silvani et al.^62^ reported that a 20% (w/w) biochar amendment reduced leachate concentrations of PFOS, PFHxS, PFOA, and PFHxA by 86%, 72%, 70%, and 31%, respectively, whereas only 10–15% reductions were observed with a 1% amendment. Sormo et al.^63^ also showed that a 0.5% dose of activated biochar reduced leaching by over 90% for PFBS, PFHxS, PFOS, PFHxA, and PFOA, though less so for PFBA (57%). At higher application rates (1–5%), leaching reductions exceeded 98% for PFBS, PFHxS, PFOS, PFHxA, and PFOA, and reached 79% for PFBA. Collectively, these results emphasize the strong dose-dependent effect of biochar in mitigating PFAS pollution across diverse soil systems.

In summary, both types of biochar demonstrated potential to reduce the water leachability of regulated PFAS in Scantic soil, with the higher-dose biochar #1 (5%) being the most effective. However, in Woodbridge soil, biochar amendments, particularly biochar #1 at a lower dose, may increase PFAS leaching. These observations are in line with our previous study with biosolids #1,^26^ where neither biochar #1 nor biochar #2 at the dose of 0.05% or 1% significantly affected the water-leachable ΣPFAS in Woodbridge soil. In Scantic soil with biosolids #1, biochar #2 at 1% significantly lowered the water-leachable ΣPFAS.^26^ Results from these two studies thus highlight the importance of site-specific investigations with respect to developing suitable biochar-based remediation strategies.

## 4 Environmental Implications and Limitations

Through comparing two types of biosolids in the same CRP-soil systems, this study revealed:

- There is a significant difference in terms of uptake of PFAAs by CRP plants, even though the concentrations of individual PFAAs are similar between biosolids #1 and #2. The decreased uptake of PFAAs observed in this study could be due to the potential toxicity of PFAS precursors presenting high concentrations in biosolids #2. Considering these results, phytoremediation may not be an effective approach for sites accepting biosolids similar to biosolids #2 when CRP species are employed.
- Given the lack of analytical tools for measuring individual PFAS precursors in biosolids #2, it is not possible to monitor their fate and distribution in the studied biosolids-soil-plant systems. It is true that we can quantify all precursors initially in the tested systems through the TOP assay, assuming that this assay can capture all precursors accurately. Using this assay, we can also quantify precursors in the water leachate from different treatments and controls. However, the difference between these two sets of values does not disclose the fraction of precursors that were transformed or the fraction of those resisting water leaching. Without knowing the extent of precursor transformation, the calculation of uptake percentage for each PFAA—the potential transformation end product—remains inaccurate. In this study, we tried to circumvent this issue by spiking two ^13^C-PFAAs. However, considering plant’s preference for ^12^C- rather than ^13^C-PFAS, the distribution of ^13^C-PFAS may not accurately represent that of native PFAS. Therefore, other approaches for tracking fate and distribution of native PFAS need to be developed.
- Understanding the role of biochar in a biosolids-soil-plant system warrants further investigation. The physicochemical properties of biochar can strongly influence its capacity to interact with PFAS and other organic and inorganic contaminants in such amended soil systems. In addition to the surface area and organic carbon content, properties such as pore structure, aromaticity, hydrophobicity, ash content, and elemental ratios (e.g., H/C and O/C) may affect interactions between biochar and PFAS with varying chain lengths and functional groups. These characteristics can influence sorption mechanisms, including hydrophobic interactions, electrostatic interactions, and pore-filling processes. While these characterizations are valuable for understanding sorption and desorption of PFAS on biochar in simple water matrices, they do not assist much in elucidating the interactions between PFAS and biochar in the soil systems. This is due to numerous factors that can potentially impact the performance of biochar toward PFAS. Such factors include abiotic components of soil and biosolids: organic matter, metal oxides, other possible contaminants, and microorganisms. Given this complexity, even if the biochar was well-characterized, it was impossible to pinpoint the exact mechanisms involved in the observed effect. To truly understand the role of biochar or other sorbent amendments toward PFAS and other pollutants, the biosolids-amended soil-plant system will need to be simplified to a certain degree. This simplification will allow thorough investigations of the potential interactions between biochar and components in such systems. Detailed mechanistic understanding from these studies can then guide the design of better amendments for target purposes.
- Understanding PFAS distribution within CRP plant tissues also deserves further study. As revealed by previous studies,^20–23^ long-chain PFAS have a higher tendency compared to short chain counterparts to accumulate in plant roots. Removing the roots can lead to the removal of PFAS from soil, which is beneficial for phytoremediation. In this study, despite multiple attempts, complete separation of fine root tissues from soil without significant loss was not possible, and therefore, the concentration and mass of PFAS in the CRP root compartments were not quantified. Improved methods for separating roots from soil (e.g., immersion in water followed by gentle brushing) are needed to allow accurate quantification of PFAS uptake by fine roots. This will lead to an accurate calculation of PFAS mass that can be removed by plant species.
- The decreased uptake of PFAS by CRP plants in soil amended with biosolids #2 may lead to the conclusion that similar biosolids, if land-applied, could bring along similar observations. Thus, their potential impact on the environment warrants further investigations. In addition, this kind of biosolids should not be applied to agricultural soils until we fully understand the composition, fate, and transformation of PFAS precursors in the biosolids. Furthermore, even with full understanding of PFAS precursors, phytoremediation with or without amendments must be evaluated at specific sites, taking into account soil properties, climate, and plant species.

## Supplementary Material



## Notes

Physiochemical properties of PFAS; information on chemicals and reagents used; LC-MS/MS instrumental parameters for PFAS analysis; characteristics of the soil; composition of the CRP (cover crop mix); appearance of the CRP mix in the greenhouse at two time points (day 33 and day 92); dry weight of the biomass and presence of PFAS and mass-labeled PFAS in grass-legume shoots; mass (ng) of Σ_13_PFAS up taken by the CRP mixture in soil amended by two types of biosolids; LOD and LOQ of PFAS; and recoveries of EIS (PDF)
