# Supplementary material for: PFAS Uptake by Plants in Soils Amended with Biosolids Derived from Wastewater with Industrial Input
Source: ACS Environ Au. 2026 Apr 21;6(4):643–53. doi: 10.1021/acsenvironau.6c00004 (PMC13377513; doi:10.1021/acsenvironau.6c00004)
Supplement: Supplementary file 1 [file vg6c00004_si_001.pdf]

Supporting Information

PFAS Uptake by Plants in Soils Amended with Biosolids Derived from Wastewater with Industrial Input

Aswin Kumar Ilango, Madhav Kharel, Weilan Zhang *, and Yanna Liang *

Department of Environmental and Sustainable Engineering, University at Albany, State University of New York, Albany, New York 12222, United States

* Corresponding authors:

Weilan Zhang: wzhang4@albany.edu

Yanna Liang: yliang3@albany.edu

Address: 1400 Washington Avenue, Albany, New York 12222, United States

Contents

Texts

Text S1. LC-MS-MS instrumental parameters, PFAS quantification, and quality control--- S3

Tables

Table S1. Chemical reagents used in this study-- S4

Table S2. Soil characteristics--- S5

Table S3. Composition of the conservation reserve program (CRP) seed mix----------- S6

Table S4. Background PFAS concentrations in the tap water used for irrigation in this study S7

Table S5. Recovery of $^{13}C_5$-PFHxA-EIS in this study versus EIS from EPA method 1633 for the quantification of target PFAAs in QC samples------------------------------ S8

Table S6. Instrumental limit of detection (LOD and limit of quantification (LOQ) for PFAS in solid matrix-- S9

Table S7. Physiochemical properties of PFAS examined in this study-------------------- S10-S12

Table S8. Two-way ANOVA for the percent removal of the detectable EPA-regulated PFAS, Σ_{13}PFAS, and the spiked isotopically labeled PFAS by CRP plant mixture over 92 days-- S13

Figures

Figure S1. Appearance of the CRP mix at two time points, day 33 and 92------------- S14

Figure S2. Dry weight of biomass at each time point and the total---------------------- S15

Figure S3. Total PFAS mass (ng) in the two cuts of the CRP exposed to (A) Kennebec sludge (Biosolids #2) and Limestone sludge (Biosolids #1) respectively--------------- S16

Text S1. LC-MS-MS instrumental parameters, PFAS quantification, and quality control.

Thirteen PFAS were quantified in the sample extracts, including perfluorocarboxylic acids (PFCAs: C_4, C_6-C_{11}), perfluorosulfonic acids (PFSAs: C_4, C_6, and C_8), GenX, 6:2 fluorotelomer sulfonate (6:2 FTS), and N-ethyl perfluorooctane sulfonamidoacetic acid (N-EtFOSAA). Isotopically labeled PFAS, ($^{13}C_3$-PFHxS and $^{13}C_9$-PFNA) spiked to biosolids, were also quantified in soil and plant samples. To correct for instrumental and matrix variability, non-extracted internal standards (NIS), perfluoro-n-[1,2,3,4-$^{13}C_4$]octanoic acid ($^{13}C_4$-PFOA) and perfluoro-n-[1,2,3,4-$^{13}C_4$]octanesulfonic acid ($^{13}C_4$-PFOS), were spiked into all samples at a concentration of 1 µg/L prior to analysis [1, 2].

Chromatographic separation was achieved using two Agilent Eclipse Plus C18 columns: a ZORBAX analytical column (3 × 50 mm, 1.8 µm) and a delay column (4.6 × 50 mm, 3.5 µm), both maintained at 50°C. The binary mobile phase consisted of solvent A (5 mM ammonium acetate in water) and solvent B (95% methanol). A gradient elution was performed at a flow rate of 0.5 mL/min, decreasing solvent A from 70% to 0% over 8 min, holding at 0% for 4 min, and re-equilibrating to the initial condition over 12 min. Analytes were detected using an Agilent triple quadrupole mass spectrometer with Jet Stream electrospray ionization (ESI) operated in negative ion mode and dynamic multiple reaction monitoring (dMRM) [1, 3, 4]. Method performance was evaluated by recovery efficiency of extracted internal standards (EIS), calculated as the ratio of measured EIS to spiked EIS concentrations [5-7]. Recoveries ranged from 70% to 130% across all sample types, confirming acceptable method precision and accuracy.

Table S1. Chemical reagents used in this study.

No.	Chemical name	Grade/purity	Procured detail
1.	Tetrabutylammonium hydrogensulfate (TBAHS)	≥98%	TCI
2.	Tert-Butyl methyl ether (MTBE)	99%	Fisher scientific
3.	Perfluorohexanoic acid	≥98%	Fisher scientific
4.	Perfluoroheptanoic acid	≥98%	Fisher scientific
5.	Perfluorooctanoic acid	≥96%	Sigma-Aldrich
6.	Perfluorononanoic acid	≥98%	Oakwood chemicals
7.	Perfluorodecanoic acid	≥98%	Matrix scientific
8.	Potassium perfluorobutanesulfonate	≥98%	Fisher scientific
9.	Perfluorohexanesulfonic acid potassium salt	≥98%	Fisher scientific
10.	Heptadecafluorooctanesulfonic acid potassium salt	≥98%	Sigma-Aldrich
11.	Undecafluoro-2-methyl-3-oxahexanoic acid	≥97%	SynQuest Laboratories
12.	6:2 Fluorotelomer sulfonic acid	≥98%	SynQuest Laboratories
13.	2-N-ethyl perfluorooctane sulfonamido acetic acid	N/A	SynQuest Laboratories
14.	Perfluoro-n-[1,2-$^{13}C_2$]octanoic acid	≥98%	Wellington Laboratories Inc.
15.	Sodium perfluoro-1[1,2,3,4-$^{13}C_4$]octanesulfonate	≥98%	Wellington Laboratories Inc.
16.	Perfluoro-n-[1,2,3,4,6-$^{13}C_5$]hexanoic acid	≥98%	Wellington Laboratories Inc.
17.	perfluoro-1-[1,2,3-$^{13}C_3$]hexanesulfonic acid	≥98%	Wellington Laboratories Inc.
18.	perfluoro-n-[$^{13}C_9$]nonanoic acid	≥98%	Wellington Laboratories Inc.
19.	Ammonium acetate	LCMS grade	Fisher scientific
20.	Methanol	LCMS/HPLC grade	Fisher scientific
21.	Ammonium hydroxide	28-30%	Fisher scientific
22.	Calcium chloride	93%	Fisher scientific
23.	Sodium hydroxide	Certified ACS	Fisher scientific
24.	Water	LCMS Grade	Fisher scientific
25.	Sodium carbonate	98%	Thermo scientific

Note: N/A: Data not available

Table S2. Soil characteristics.

Soil properties	Woodbridge series	Scantic series
Texture	Silt loam (sand: 26%; silt: 58%; clay: 15%)	Silty clay loam (sand: 7%; silt: 59%; clay: 33%)
Predicted available water capacity (g/g)	0.25	0.28
Aggregate stability (%)	58.50	35.60
Organic matter (%)	Organic carbon: 3.09; total carbon: 3.10; total nitrogen: 0.23	Organic carbon: 2.27; total carbon: 2.29; total nitrogen: 0.21
Autoclaved citrate extractable (ACE) soil protein index	13.6	6.7
Soil respiration (mg)	0.5	0.8
Biological active carbon (mg/L)	472	526
Soil pH	5.9	5.9
Extractable phosphorus (mg/L)	3.4	2.9
Extractable potassium (mg/L)	36	157.4
Additional Nutrients (mg/L)	Ca: 471.9; Mg: 45.4; S: 15.5; Al: 260.7; B: 0.02; Cu: 0.25; Fe: 13.1; Mn: 10.2; Zn: 0.4	Ca: 1045.5; Mg: 143.4; S: 9.1; Al: 89.8; B: 0.01; Cu: 0.17; Fe: 28.8; Mn: 12.2; Zn: 0.5

Table S3. Composition of the conservation reserve program (CRP) seed mix.

	Seeding Rate, lb/acre, seeded in mixture	% in mixture
**Grasses**		
Kentucky Bluegrass	10	19.23
Timothy grass	6	11.54
Orchardgrass	3	5.77
Smooth Bromegrass	6	11.54
Total		*48.08*
**Legumes**		
Alfalfa	10	19.23
Red Clover	4	7.69
White Clover	4	7.69
Alsike Clover	5	9.62
Birdsfoot Trefoil	4	7.69
Total		*51.92*

Table S4. Background PFAS concentrations in the tap water used for irrigation in this study. It needs to be noted that the LOQ in the tap water is 100 times less than the instrument LOQ since 500 mL of tap water was passed through the SPE cartridge and the final elution volume was 5 mL. This was in line with the procedure detailed in EPA Method 1633A.

**PFAS tested**	**Tap water (ng/L)**	**Instrument (ng/L)**	
		**LOD**	**LOQ**
PFBA	< LOD	39.1	78.1
PFPeA	< LOD	19.5	39.1
PFHxA	< LOD	19.5	39.1
PFHpA	< LOD	19.5	39.1
PFOA	< LOD	39.1	78.1
PFNA	< LOD	39.1	78.1
PFDA	< LOD	19.5	39.1
PFUnA	< LOD	19.5	39.1
PFBS	< LOD	4.88	9.77
PFHxS	< LOD	19.5	39.1
PFOS	< LOD	19.5	39.1
6:2 FTS	< LOD	39.1	78.1
NEtFOSAA	< LOD	19.5	39.1
HFPO-DA	< LOD	39.1	78.1

Note: LOD is limit of detection and LOQ is limit of quantification.

Table S5. Recovery of $^{13}C_5$-PFHxA-EIS in this study versus EIS from EPA method 1633 for the quantification of target PFAAs in QC samples.

**EIS used in this study**						**EIS from EPA method 1633**					
**EIS**	**Mass added (ng)**	**Target PFAS**	**Average EIS recovery (%) in QC samples**			**EIS mixtures**	**Mass added (ng)**	**Targeting PFAS**	**Average EIS recovery (%) in QC samples**		
			**Matrix blank**	**LLOPR**	**MLOPR**				**Matrix blank**	**LLOPR**	**MLOPR**
$^{13}C_5$-PFHxA	10	PFBA	98.5 ± 0.04	98.5 ± 0.12	98.9 ± 0.04	$^{13}C_4$-PFBA	40	PFBA	99.9 ± 0.13	97.1 ± 0.97	99.8 ± 0.99
$^{13}C_5$-PFHxA	10	PFPeA	99.4 ± 0.05	98.9 ± 0.45	99.9 ± 0.50	$^{13}C_5$-PFPeA	20	PFPeA	99.4 ± 0.56	99.3 ± 0.98	101 ± 0.01
$^{13}C_5$-PFHxA	10	PFHxA	100 ± 0.17	99.9 ± 0.32	102 ± 0.25	$^{13}C_5$-PFHxA	10	PFHxA	98.6 ± 1.12	100 ± 1.01	99.7 ± 1.05
$^{13}C_5$-PFHxA	10	PFHpA	100 ± 0.51	99.1 ± 0.58	100 ± 0.22	$^{13}C_4$-PFHpA	10	PFHpA	99.9 ± 0.21	96.7 ± 0.97	101 ± 1.06
$^{13}C_5$-PFHxA	10	PFOA	95.6 ± 0.04	98.0 ± 0.51	99.6 ± 0.23	$^{13}C_8$-PFOA	10	PFOA	98.7 ± 0.14	96.7 ± 0.97	99.6 ± 1.05
$^{13}C_5$-PFHxA	10	PFNA	94.7 ± 0.04	102 ± 0.51	102 ± 0.62	$^{13}C_9$-PFNA	5	PFNA	99.3 ± 0.07	98.2 ± 0.98	98.2 ± 0.98
$^{13}C_5$-PFHxA	10	PFDA	94.8 ± 0.08	100 ± 0.27	98.9 ± 0.08	$^{13}C_6$-PFDA	5	PFDA	99.9 ± 0.05	96.6 ± 0.97	99.7 ± 0.99
$^{13}C_5$-PFHxA	10	PFUnA	98.6 ± 0.11	99.2 ± 0.06	99.8 ± 0.29	$^{13}C_7$-PFUnA	5	PFUnA	101 ± 0.13	98.8 ± 0.99	99.6 ± 0.99
$^{13}C_5$-PFHxA	10	PFBS	101 ± 0.31	99.8 ± 0.28	98.9 ± 0.25	$^{13}C_3$-PFBS	10	PFBS	98.6 ± 0.24	99.6 ± 0.99	98.5 ± 0.99
$^{13}C_5$-PFHxA	10	PFHxS	98.9 ± 0.04	99.7 ± 0.05	99.2 ± 0.05	$^{13}C_3$-PFHxS	10	PFHxS	98.6 ± 0.15	98.3 ± 0.98	99.3 ± 0.99
$^{13}C_5$-PFHxA	10	PFOS	97.4 ± 0.11	98.1 ± 0.14	98.6 ± 0.15	$^{13}C_3$-PFOS	12.5	PFOS	102 ± 0.57	99.0 ± 0.99	98.2 ± 0.98
$^{13}C_5$-PFHxA	10	6:2 FTS	94.1 ± 0.12	98.6 ± 0.21	98.0 ± 0.08	$^{13}C_2$-6:2 FTS	20	6:2 FTS	100 ± 0.45	96.4 ± 0.96	99.5 ± 0.99
$^{13}C_5$-PFHxA	10	NEtFOSAA	94.3 ± 0.68	97.9 ± 0.34	95.4 ± 0.29	D_5-NEtFOSAA	20	NEtFOSAA	98.5 ± 0.51	97.2 ± 0.97	99.23 ± 0.99
$^{13}C_5$-PFHxA	10	HFPO-DA	97.3 ± 0.11	102 ± 1.06	99.6 ± 0.10	$^{13}C_3$-HFPO-DA	40	HFPO-DA	99.0 ± 0.07	97.2 ± 0.97	103 ± 1.02

Note: Quality control (QC) samples, including method blanks using Ottawa sand; low level ongoing precision and recovery standards (LLOPR), and mid-level OPR (MLOPR) using the CPR shoots from controls without exposure to PFAS.

Table S6. Instrumental limit of detection (LOD) and limit of quantification (LOQ) for PFAS in solid matrix.

PFAS tested	Solid matrix			
	Instrument (ng/L)		Samples (ng/L)	
	**LOD**	**LOQ**	**LOD**	**LOQ**
PFBA	39.1	78.1	0.04	0.08
PFPeA	19.5	39.1	0.02	0.04
PFHxA	19.5	39.1	0.02	0.04
PFHpA	19.5	39.1	0.02	0.04
PFOA	39.1	78.1	0.04	0.08
PFNA	39.1	78.1	0.04	0.08
PFDA	19.5	39.1	0.02	0.04
PFUnA	19.5	39.1	0.02	0.04
PFBS	4.88	9.77	0.005	0.01
PFHxS	19.5	39.1	0.02	0.04
PFOS	19.5	39.1	0.02	0.04
6:2 FTS	39.1	78.1	0.04	0.08
NEtFOSAA	19.5	39.1	0.02	0.04
HFPO-DA	39.1	78.1	0.04	0.08

Table S7. Physiochemical properties of PFAS examined in this study.

No.	Category	Compound name	Chemical structure	Chemical formula	Molecular weight (g/mol)	S_w (25 °C) (g/L)	$_{P}K_a$ (25 °C)
1.	Short-chain PFCA	Perfluorobutanoic acid (PFBA)	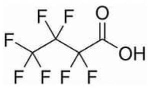	$C_4HF_7O_2$	214.1	N/A	0.4[8]
2.	Short-chain PFCA	Perfluorohexanoic acid (PFHxA)	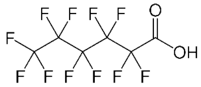	$C_6HF_{11}O_2$	314	15.7 [9]	-0.16 [8]
3.	Short-chain PFCA	Perfluoroheptanoic acid (PFHpA)	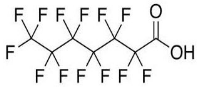	$C_7HF_{13}O_2$	364	3.65×10^{-3} [10]	−2.29[10]
4.	Long-chain PFCA	Perfluorooctanoic acid (PFOA)	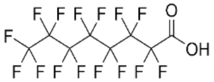	$C_8HF_{15}O_2$	414	3.4 [9]	-0.2[8]
5.	Long-chain PFCA	Perfluorononanoic acid (PFNA)	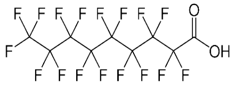	$C_9HF_{17}O_2$	464	6.25×10^{-2} [10]	-0.21[10]
6.	Long-chain PFCA	Perfluorodecanoic acid (PFDA)	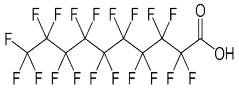	$C_{10}HF_{19}O_2$	514	9.5 [11]	-5.2[12]
7.	Long-chain PFCA	Perfluoroundecanoic acid (PFUnA)	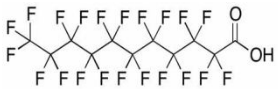	$C_{11}HF_{21}O_2$	564	4×10^{-3} [11]	-5.2[12]

8.	Short-chain PFSA	Potassium perfluorobutane sulfonate (PFBS)	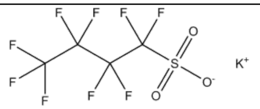	$C_4F_9O_3SK$	338	46.2 [13]	0.14[8]
9.	Long-chain PFSA	Perfluorohexanesulfonic acid potassium salt (PFHxS)	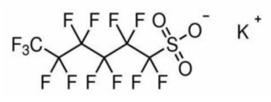	$C_6F_{13}KO_3SK$	438	2.3[11]	0.14[8]
10.	Long-chain PFSA	Heptadecafluorooctane sulfonic acid potassium salt (PFOS)	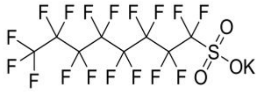	$C_8HF_{17}KO_3SK$	538	0.57[9]	-3.27[14]
11.	PFOA alternative	Undecafluoro-2-methyl-3-oxahexanoic acid (GenX)	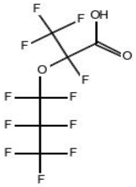	$C_6HF_{11}O_3$	330.05	N/A	2.84[12]
12.	Precursor	6:2 fluorotelomer sulfonic acid (6:2 FTS)	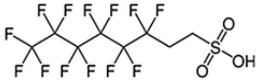	$C_8H_5F_{13}O_3S$	428	1.3[11]	1.31[11]
13.	Precursor	2-*N*-ethyl perfluorooctane sulfonamido acetic acid (N-EtFOSAA)	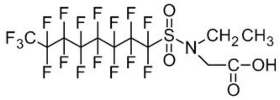	$C_{12}H_8F_{17}NO_4S$	585	N/A	N/A
14	Mass labeled	Sodium perfluoro-1-[1,2,3-$^{13}C_3$]hexanesulfonate (M3PFHxS)	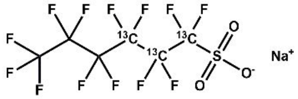	$C_6F_{13}NaO_3S$	425.08	N/A	N/A

15	Mass labeled	Perfluoro-n-[1,2,3,4,5-$^{13}C_5$]nonanoic acid (MPFNA)	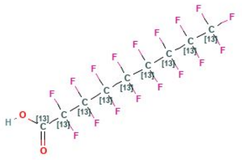	$C_9HF_{17}O_2$	473.010	N/A	N/A

Note: Solubility in water (S_w); Dissociation constant ($_pK_a$); N/A: Data not available

Table S8. Two-way ANOVA for the percent removal of the detectable EPA-regulated PFAS, Σ_{13}PFAS, and the spiked isotopically labeled PFAS by CRP plant mixture over 92 days.

	Dependent Variable	Woodbridge soil *p* value	Scantic soil *p* value
Biochar	PFOA	0.01	0.406
	PFNA	0.985	0.14
	PFHxS	0.15	1
	PFOS	0.837	0.524
	$^{13}C_3$-PFHxS	0.031	0
	$^{13}C_9$-PFNA	0.01	0.824
	Σ_{13}PFAS	0.012	0.203
dose	PFOA	0.219	0.413
	PFNA	0.152	0.754
	PFHxS	0.15	1
	PFOS	0.97	0.445
	$^{13}C_3$-PFHxS	0.001	0
	$^{13}C_9$-PFNA	0.009	0.014
	Σ_{13}PFAS	0.701	0.195
Biochar * dose	PFOA	0.094	0.334
	PFNA	0.128	0.629
	PFHxS	0.15	1
	PFOS	0.106	0.421
	$^{13}C_3$-PFHxS	0	0.001
	$^{13}C_9$-PFNA	0	0.644
	Σ_{13}PFAS	0.002	0.198

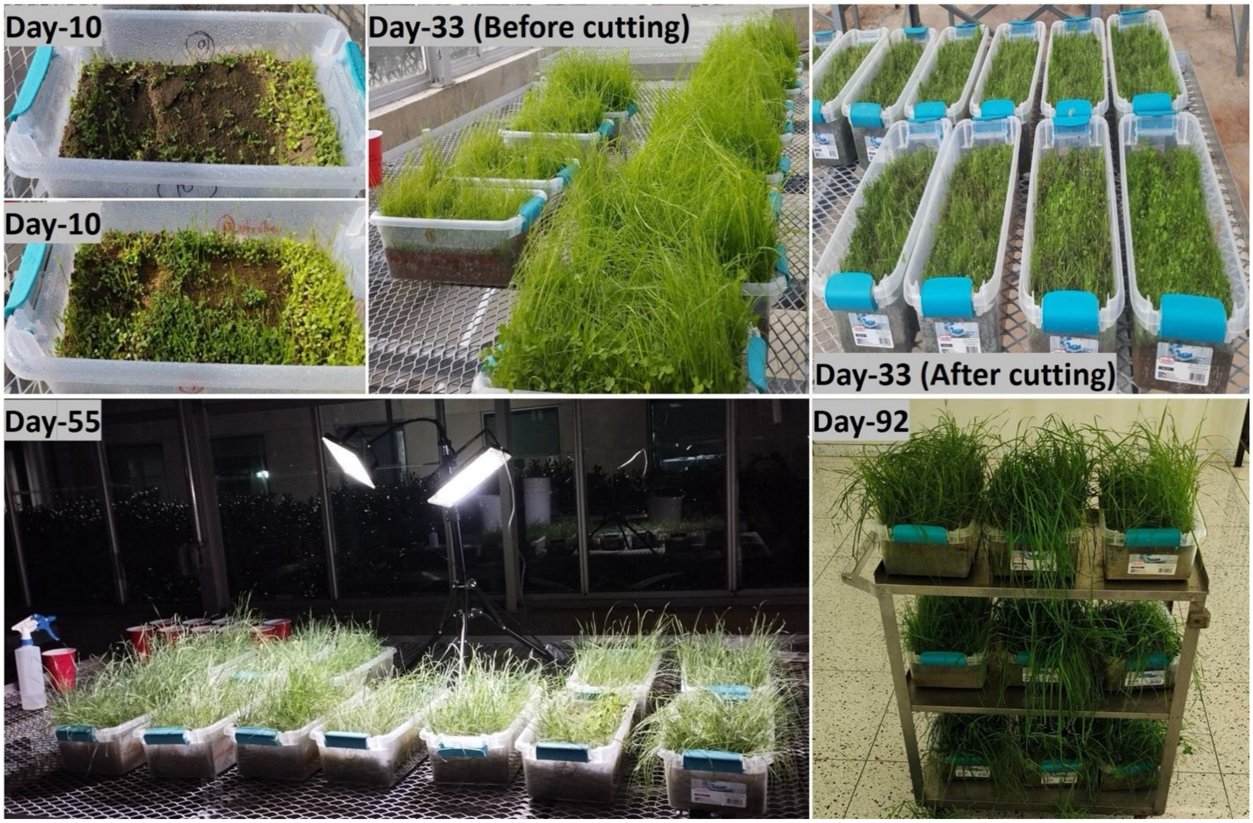

Figure S1. Appearance of the CRP mix at two time points, day 33 and 92.

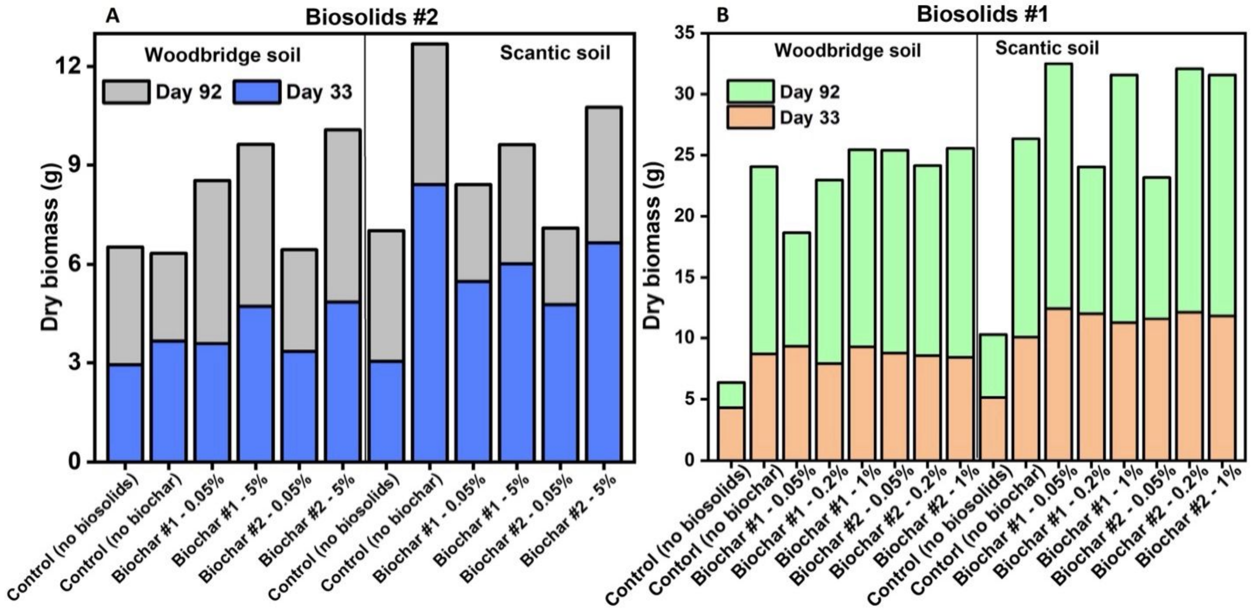

Figure S2. Dry weight of biomass at each time-point and total dry weight of biomass at harvest by soils amended with (A) Biosolids #2 and (B) Biosolids #1, respectively.

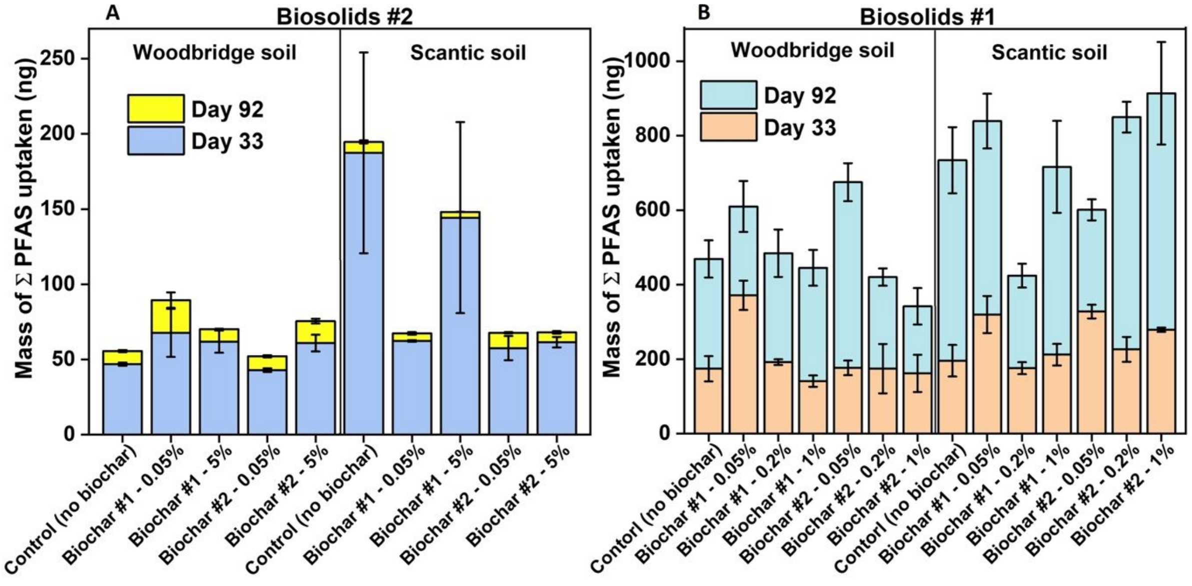

Figure S3. Total PFAS mass (ng) in the two cuts of the CRP exposed to (A) Kennebec sludge (Biosolids #2) and Limestone sludge (Biosolids #1) respectively.

References

(1) Zhang, W.; Zhang, D.; Liang, Y. Nanotechnology in remediation of water contaminated by poly-and perfluoroalkyl substances: A review. *Environmental pollution* **2019**, *247*, 266-276.
(2) Zhang, W.; Zhang, Q.; Liang, Y. Ineffectiveness of ultrasound at low frequency for treating per-and polyfluoroalkyl substances in sewage sludge. *Chemosphere* **2022**, *286*, 131748.
(3) Zhang, W.; Cao, H.; Mahadevan Subramanya, S.; Savage, P.; Liang, Y. Destruction of Perfluoroalkyl Acids Accumulated in Typha latifolia through Hydrothermal Liquefaction. *ACS Sustainable Chemistry & Engineering* **2020**, *8* (25), 9257-9262.
(4) Zhang, W.; Zhang, Q.; Liang, Y. Ineffectiveness of ultrasound at low frequency for treating per- and polyfluoroalkyl substances in sewage sludge. *Chemosphere* **2022**, *286*, 131748. DOI: https://doi.org/10.1016/j.chemosphere.2021.131748.
(5) Zhang, W.; Wellington, T. E.; Liang, Y. Effect of two sorbents on the distribution and transformation of N-ethyl perfluorooctane sulfonamido acetic acid (N-EtFOSAA) in soil-soybean systems. *Environmental Pollution* **2023**, *318*, 120941. DOI: https://doi.org/10.1016/j.envpol.2022.120941.
(6) Zhang, W.; Liang, Y. Changing bioavailability of per- and polyfluoroalkyl substances (PFAS) to plant in biosolids amended soil through stabilization or mobilization. *Environmental Pollution* **2022**, *308*, 119724. DOI: https://doi.org/10.1016/j.envpol.2022.119724.
(7) Zhang, W.; Liang, Y. Performance of different sorbents toward stabilizing per- and polyfluoroalkyl substances (PFAS) in soil. *Environmental Advances* **2022**, *8*, 100217. DOI: https://doi.org/10.1016/j.envadv.2022.100217.
(8) Steinle-Darling, E.; Reinhard, M. Nanofiltration for trace organic contaminant removal: structure, solution, and membrane fouling effects on the rejection of perfluorochemicals. *Environmental science & technology* **2008**, *42* (14), 5292-5297.
(9) Fujii, S.; Polprasert, C.; Tanaka, S.; Hong Lien, N. P.; Qiu, Y. New POPs in the water environment: distribution, bioaccumulation and treatment of perfluorinated compounds–a review paper. *Journal of Water Supply: Research and Technology—AQUA* **2007**, *56* (5), 313-326.
(10) Kim, S.; Chen, J.; Cheng, T.; Gindulyte, A.; He, J.; He, S.; Li, Q.; Shoemaker, B. A.; Thiessen, P. A.; Yu, B.; et al. PubChem in 2021: new data content and improved web interfaces. *Nucleic Acids Research* **2021**, *49* (D1), D1388-D1395. DOI: 10.1093/nar/gkaa971 (acccessed 2/22/2023).
(11) Christensen, E. R.; Wang, Y.; Huo, J.; Li, A. Properties and fate and transport of persistent and mobile polar organic water pollutants: A review. *Journal of Environmental Chemical Engineering* **2022**, 107201.
(12) Pauletto, P. S.; Bandosz, T. J. Activated carbon versus metal-organic frameworks: A review of their PFAS adsorption performance. *Journal of Hazardous Materials* **2022**, *425*, 127810.
(13) Zhou, Q.; Deng, S.; Yu, Q.; Zhang, Q.; Yu, G.; Huang, J.; He, H. Sorption of perfluorooctane sulfonate on organo-montmorillonites. *Chemosphere* **2010**, *78* (6), 688-694.
(14) Brooke, D.; Footitt, A.; Nwaogu, T. Environmental risk evaluation report: Perfluorooctanesulphonate (PFOS). **2004**.
